# Sigmoid volvulus and incidental enterobiasis in a young adult: a case report

**DOI:** 10.3389/fsurg.2026.1883256

**Published:** 2026-07-06

**Authors:** Majed Yahya M. Alshahrani, Fares Rayzah, Ibrahim M. ALmanjahi, Abdullah A. Alquzi, Hisham Sabry Abdelhafiz, Yahya Shabi

**Affiliations:** 1 Department of General Surgery, Aseer Central Hospital, Abha, Saudi Arabia; 2 Department of Internal Medicine, Aseer Central Hospital, Abha, Saudi Arabia; 3 Department of Radiology, Aseer Central Hospital, Abha, Saudi Arabia; 4 Department of Microbiology and Clinical Parasitology, College of Medicine, King Khalid University, Abha, Saudi Arabia

**Keywords:** case report, colonoscopic detorsion, *Enterobius vermicularis*, laparoscopic sigmoidectomy, sigmoid volvulus, whirl sign, young adult

## Abstract

**Background:**

Sigmoid volvulus is a disease that typically occurs in elderly patients with chronic constipation and an elongated sigmoid mesentery. Its occurrence in previously healthy young adults is uncommon, often with delayed diagnosis, and may coexist with under-recognized predisposing factors. In this case, family clustering of vague gastrointestinal symptoms and a coexisting parasitic infestation are most likely incidental rather than causative.

**Case presentation:**

A previously healthy young Saudi woman presented with 2 weeks of intermittent colicky abdominal pain, low-volume diarrhea, and a final acute episode of severe pain with progressive abdominal distension. Her 15-year-old sister had recently been diagnosed with enterobiasis with a similar symptom pattern. Vital signs were stable; abdominal examination showed mild distension without peritonism. Plain radiography demonstrated dilated colonic loops, and contrast-enhanced computed tomography revealed a markedly dilated sigmoid colon (up to 85 mm), an abrupt collapse point, and the whirl sign, consistent with sigmoid volvulus. Endoscopic detorsion was achieved successfully with placement of a rectal decompression tube; during colonoscopy, a single adult gravid *Enterobius vermicularis* was retrieved and confirmed parasitologically (characteristic ova demonstrated within the gravid female), prompting consultation with an infectious diseases team and a course of mebendazole. Following clinical stabilization, the patient underwent laparoscopic sigmoidectomy with extracorporeal stapled colorectal anastomosis. Operative findings confirmed a redundant sigmoid colon without ischemia, perforation, or contamination. Histopathology revealed ischemic-type changes (mucosal atrophy, submucosal edema, congested vessels, and hypertrophied muscularis propria) with viable resection margins. At the 4-week follow-up, she was asymptomatic and repeat parasitological testing (stool microscopy and adhesive tape examination) showed no *E. vermicularis* ova.

**Conclusion:**

Sigmoid volvulus should be considered in young adults presenting with acute colonic obstruction, particularly when imaging features are characteristic. A two-stage strategy of colonoscopic detorsion followed by definitive laparoscopic sigmoidectomy is feasible and effective in hemodynamically stable patients without ischemia. The clinical relevance of the coexisting enterobiasis remains hypothesis-generating and should not be overstated.

## 1 Introduction

Sigmoid volvulus is the third most common cause of large-bowel obstruction worldwide and results from torsion of a redundant sigmoid colon around a narrow mesenteric base, producing a closed-loop obstruction that may progress to ischemia, gangrene, perforation, or peritonitis if not promptly relieved (1). It is classically described in elderly, institutionalized, or neuropsychiatrically impaired patients, although a younger age profile is recognized in endemic “volvulus-belt” populations (1).

In previously healthy young adults outside these endemic settings, sigmoid volvulus is uncommon, reported mainly as isolated cases, and frequently subject to diagnostic delay because the presentation is non-specific and the diagnosis is rarely considered first (2–6). Contrast-enhanced computed tomography (CT) is the diagnostic gold standard: the whirl sign and coffee-bean configuration are highly suggestive, and CT also assesses ischemia and perforation, which determine whether endoscopic or immediate operative management is appropriate (1, 4).

We report a previously healthy young Saudi woman with CT-confirmed sigmoid volvulus managed by colonoscopic detorsion followed by elective laparoscopic sigmoidectomy, in whom a parasitologically confirmed *Enterobius vermicularis* adult was retrieved incidentally during endoscopy against a background of recently diagnosed enterobiasis in her younger sister. The case is reported for the following three reasons: the unusual young age without classical risk factors, the incidental but confirmed enterobiasis, and the successful stepwise endoscopic-to-laparoscopic pathway with histologically viable margins. The operative decision-making is examined in detail in the Discussion section, and throughout we distinguish proven facts from plausible but unconfirmed associations and avoid attributing causality to the parasitic finding (7–9).

## 2 Case presentation

### 2.1 Demographics and history

A previously healthy 24-year-old Saudi woman was transferred from a regional hospital with a working diagnosis of sigmoid volvulus. Her past medical and surgical history was unremarkable, and she reported no chronic constipation, neurological or psychiatric disorders, prior abdominal surgery, or use of psychotropic or anticholinergic medications. There were no known drug allergies.

She described approximately 2 weeks of intermittent colicky abdominal pain associated with low-volume diarrhea, bloating, and difficulty defecating. On the morning of the day before admission, the pain recurred abruptly, was colicky in character, progressively worsened through the night, and reached a self-reported intensity of 10/10 by the time of presentation, with associated nausea but no vomiting and increasing abdominal distension. She passed loose stool once during the preceding night and had no further bowel motion thereafter. The patient subjectively reported fever; however, her measured admission temperature was within normal limits (afebrile).

Of note, her 15-year-old sister had presented 1 month earlier with a similar pattern of intermittent abdominal pain, bloating, diarrhea, and difficult defecation persisting for approximately 1 year, and had been diagnosed with *E. vermicularis* infection at another hospital on the basis of microscopic identification; her symptoms reportedly resolved after a course of mebendazole. There was no other relevant family history of inflammatory bowel disease, colorectal malignancy, congenital colonic anomalies, or known hereditary motility disorders.

### 2.2 Physical examination

On admission, the patient was alert and oriented, with stable vital signs [hemodynamically stable, peripheral oxygen saturation within normal limits, and admission temperature within normal limits (afebrile)]. Abdominal examination revealed mild distension, predominantly in the epigastric and supraumbilical regions, without surgical scars or skin pigmentation. There was mild tenderness in the supraumbilical and epigastric areas and in the left upper quadrant but no tenderness or distension in the right upper, right lower, or left lower quadrants, and no signs of peritonism. Bowel sounds were normal in character. Digital rectal examination demonstrated an empty rectum.

### 2.3 Laboratory investigations

Initial venous blood gas analysis showed a mildly alkaline pH of 7.44, partial pressure of carbon dioxide (pCO₂) of 25 mmHg, sodium 135 mmol/L, potassium 2.8 mmol/L (with later serum potassium of 3.45 mmol/L), bicarbonate 17 mmol/L, and lactate 1 mmol/L, consistent with a mixed mild metabolic acidosis and compensatory hyperventilation, and clinically significant hypokalemia requiring electrolyte correction. The hypokalemia (2.8 mmol/L) was attributed to the preceding 2 weeks of low-volume diarrhea and reduced oral intake, compounded by fluid and electrolyte sequestration within the closed-loop obstruction; it was regarded as a consequence rather than a cause of the volvulus, although it may have aggravated the colonic dysmotility, and it was corrected before endoscopic and surgical intervention. Complete blood count revealed a total leucocyte count of 7.41 × 10⁹/L with marked neutrophilic predominance (88%) and relative lymphopenia (9%); hemoglobin was 13.6 g/dL and platelets 326 × 10⁹/L. Renal profile demonstrated creatinine 0.61 mg/dL and urea 27.82 mg/dL. Random glucose was 137 mg/dL. Coagulation parameters were within acceptable limits (International Normalized Ratio 1.22, prothrombin time 16.2 s, and activated partial thromboplastin time 32.2 s). Screening for human immunodeficiency virus and hepatitis C antibody was non-reactive. Stool culture isolated neither *Salmonella* nor *Shigella*, and stool microscopy demonstrated black, watery stool without mucus, with 2–5 red blood cells/high power field (HPF), 2–5 white blood cells/HPF, and +1 yeast forms. Parasitological examination was performed to support the clinical suspicion of pinworm infection raised by endoscopic visualization and the family history (7, 8): stool microscopy at presentation did not demonstrate *E. vermicularis* ova (a recognized limitation of stool examination for this parasite, which is more reliably identified by perianal adhesive-tape testing). The worm retrieved at colonoscopy was submitted for parasitological examination; macroscopic examination identified a white, thread-like adult helminth consistent with *E. vermicularis*, and microscopic examination of the worm compressed between two glass slides demonstrated numerous characteristic *E. vermicularis* ova within the gravid female, confirming an adult gravid *E. vermicularis* (pinworm).

### 2.4 Imaging

A plain supine abdominal radiograph demonstrated a dilated bowel loop with the gas pattern suggestive of large-bowel distension. Contrast-enhanced multidetector CT of the abdomen and pelvis (performed at the referring hospital) demonstrated marked segmental dilation of the sigmoid colon (diameter up to 85 mm), an abrupt point of bowel collapse at the volvulus, and a clearly identifiable whirl sign of twisted mesenteric vessels (Figures 1A–C). There was no small-bowel dilation, no pneumatosis intestinalis, no portal venous gas, and no free intraperitoneal air. The remainder of the bowel and the solid abdominal viscera were unremarkable. The reporting radiologist categorized the study as abnormal-urgent, with an impression highly suggestive of sigmoid colon volvulus.

**Figure 1:**
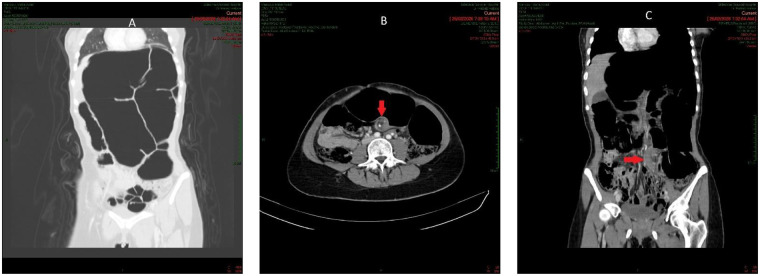
Cross-sectional imaging of the sigmoid volvulus. **(A)** Coronal contrast-enhanced CT of the abdomen and pelvis shows marked dilatation of the sigmoid colon (maximum diameter of approximately 85 mm), with an inverted-U/coffee-bean appearance. **(B)** Axial CT image at the level of the sigmoid mesentery shows the whirl sign produced by the twisting of the sigmoid mesenteric vessels around a central axis (arrow). **(C)** Coronal CT image demonstrates the abrupt point of bowel collapse at the volvulus transition zone, with proximal large-bowel dilatation and a relatively collapsed distal sigmoid and rectum. All identifying patient information, hospital identifiers, accession numbers, dates of birth, and machine-specific tags have been redacted.

### 2.5 Initial management and endoscopic detorsion

The patient was admitted under the general surgical service, kept nil per os, and administered crystalloid intravenous fluids and electrolyte replacement (particularly potassium). Empirical broad-spectrum intravenous antibiotic prophylaxis (cefuroxime 750 mg every 8 h and metronidazole 500 mg every 8 h) was initiated, together with subcutaneous unfractionated heparin 5,000 IU twice daily for venous thromboembolism prophylaxis, intravenous metoclopramide as needed for nausea, and intravenous omeprazole 40 mg once daily. Mebendazole 100 mg orally twice daily was initiated empirically as later detailed.

After a multidisciplinary discussion and provision of informed consent, the patient was transferred to the endoscopy suite for colonoscopic decompression and detorsion. The unprepared colonoscopy demonstrated normal-appearing mucosa of the distal colon and rectum; the scope was advanced beyond the level of the volvulus, where successful detorsion was performed with prompt decompression of gas and feculent fluid. A rectal decompression tube was secured under endoscopic guidance, and no immediate complication (such as perforation, bleeding, or vasovagal reaction) was encountered. During the procedure, an intraluminal worm consistent in macroscopic appearance with an adult gravid *E. vermicularis* was visualized (Figure 2). The endoscopic findings were therefore recorded as follows: successful detorsion of sigmoid volvulus, healthy-appearing mucosa beyond the obstruction (with no features of mucosal ischemia, gangrene, or pseudomembrane), and a single intraluminal worm of uncertain species but clinically suspected to be a pinworm.

**Figure 2:**
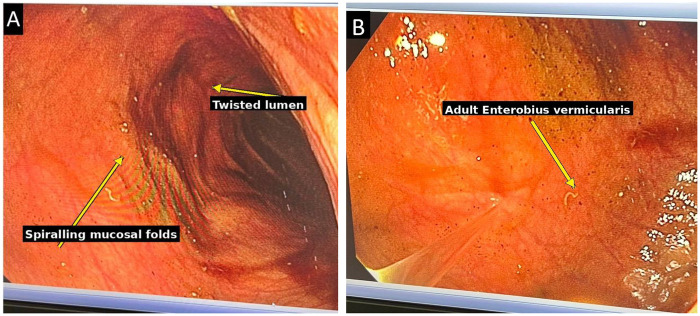
Endoscopic findings during colonoscopic detorsion. **(A)** Twisted sigmoid lumen with concentric mucosal folds spirals toward the volvulus point (arrows). **(B)** The intraluminal thin white linear structure (arrow) is macroscopically consistent with an adult gravid *E. vermicularis*.

### 2.6 Assessment of the infectious diseases team

The infectious diseases team considered the endoscopic finding most consistent with enterobiasis, particularly considering the family history of confirmed pinworm infection in a sibling with overlapping symptoms (7–9). The plan comprised the following: oral mebendazole 100 mg twice daily for 3 days (with consideration of a repeat dose at 2 weeks if symptoms recurred); submission of stool samples for routine microscopy and culture; and a recommendation to consider empirical treatment for symptomatic household contacts and screening of asymptomatic close contacts, consistent with published management of household enterobiasis clusters (7).

### 2.7 Definitive surgery

Following clinical stabilization, normalization of electrolytes, and confirmation of a viable colon on endoscopy, the patient was scheduled for definitive elective laparoscopic sigmoidectomy during the same admission, consistent with current World Society of Emergency Surgery (WSES) guidance (1, 10). Surgery was performed on Day 6 after colonoscopic detorsion. After confirmation of informed consent, the patient was placed supine in a modified lithotomy position under general endotracheal anesthesia. Pneumoperitoneum was established using the open Hasson technique at the umbilicus; a 10-mm camera port was placed at the umbilicus and two 5-mm working ports were inserted under direct vision in the right and left lower quadrants. Diagnostic laparoscopy revealed a redundant sigmoid colon consistent with prior volvulus, without macroscopic evidence of ischemia, perforation, or gross peritoneal contamination (Figure 3).

**Figure 3:**
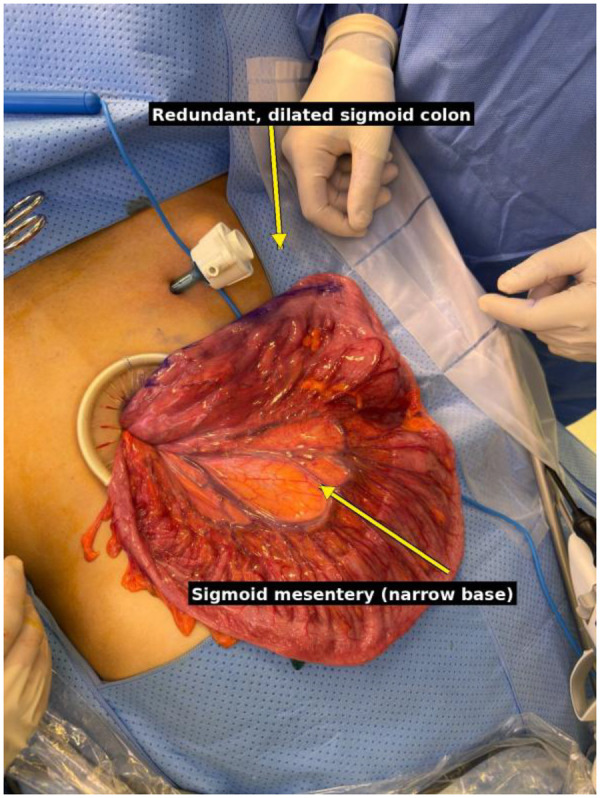
Intraoperative appearance of the redundant sigmoid colon. This intraoperative photograph obtained during laparoscopic-assisted sigmoidectomy shows a markedly redundant and dilated sigmoid colon, delivered extracorporeally through a protected mini-laparotomy incision. Arrows indicate the redundant, dilated sigmoid colon and its long, narrow-based mesentery. The bowel appears viable, with no gross evidence of perforation, transmural ischemia, or peritoneal contamination. The long mesenteric attachment is consistent with the anatomical predisposition underlying sigmoid volvulus. All identifying patient information and operative tags have been removed.

The sigmoid colon was mobilized laparoscopically to obtain an adequate length for resection and a tension-free anastomosis. The intended distal resection margin was marked intracorporeally. A small extension incision was made and an Alexis wound protector inserted; the sigmoid loop was delivered extracorporeally (Figure 3), and segmental resection was performed. A stapled colorectal anastomosis was fashioned using a linear cutting stapler. Anastomotic integrity was confirmed visually, with good serosal perfusion and absence of leaks on intraoperative inspection. After returning the bowel to the peritoneal cavity, re-establishing pneumoperitoneum, and performing a final inspection, the working ports were removed under vision, the fascia was closed with non-absorbable monofilament sutures, and the skin was closed with staples. The patient was extubated in the operating theater and transferred to the recovery area in a stable condition.

### 2.8 Histopathology

The fixed specimen consisted of a 35-cm segment of colon entirely covered by mesentery, with a dark-brown serosal surface; the lumen measured 5–6 cm at each stapled end. The attached mesentery measured 15 cm × 4.5 cm × 0.7 cm. Sectioning revealed a congested and focally flattened mucosa. Microscopic examination showed focally atrophic colonic mucosa overlying edematous submucosa and edematous subserosal fat containing scattered congested vessels. The muscularis propria was moderately hypertrophied. The attached mesentery was edematous, with dilated and congested vessels and two small reactive lymph nodes. Both surgical resection margins were viable. The integrated pathological diagnosis was as follows: “colon with features of ischemic changes (edematous wall and dilated, congested blood vessels) consistent with sigmoid volvulus; viable surgical margins.” No transmural necrosis, perforation, or dysplasia was identified, and no parasitic organisms were observed within the surgical specimen on routine hematoxylin and eosin sections. Ganglion cells were present in the myenteric plexus, and there was no histological evidence of aganglionosis, neuronal intestinal dysplasia, or other intrinsic neuromuscular abnormality, nor of eosinophilic or granulomatous inflammation, effectively excluding an underlying motility disorder as a cause of the volvulus. Photomicrographs of the specimen were not available for inclusion in this report.

## 3 Differential diagnosis

The differential diagnoses considered and excluded included colonic pseudo-obstruction (Ogilvie syndrome), distal mechanical obstruction due to an obstructing left-sided colorectal neoplasm, complicated diverticular disease with stricture, Hirschsprung-related megacolon, foreign-body or bezoar obstruction, and toxic megacolon. Imaging, intraoperative inspection, and histopathology effectively excluded each of these in turn.

## 4 Follow-up and outcomes

On the first postoperative day, the patient was alert, oriented, and ambulating. She reported one episode of vomiting on the evening of surgery but no subsequent nausea, abdominal pain, or respiratory symptoms. Pulse was 85 beats/min, respiratory rate 19/min, blood pressure 127/75 mmHg, and tympanic temperature 36.7°C. The abdomen was soft and non-tender, and the surgical wound dressings were dry and intact. She started on a clear-liquid diet on postoperative Day 1, advanced to a soft diet after passage of flatus and small-volume bowel motions, and was discharged tolerating a normal diet without complication. At outpatient follow-up in the general-surgery clinic at 4 weeks postoperatively, she was asymptomatic, with a soft, non-distended abdomen and clean, dry wounds; she was subsequently discharged from surgical follow-up. The Clavien–Dindo grade of the postoperative course was Grade I. No recurrence, anastomotic complication, surgical site infection, or readmission was documented during the 4-week follow-up period. With regard to the enterobiasis, repeat parasitological testing at the 4-week visit (stool microscopy and perianal adhesive tape examination) showed no *E. vermicularis* ova, and the patient reported no recurrent perianal pruritus or gastrointestinal symptoms, consistent with parasitological clearance following mebendazole therapy. The patient and family received counseling regarding hand hygiene, laundering of bed linen and underwear in hot water, and household treatment of enterobiasis, in line with current parasitology and public health recommendations (7–9).

## 5 Discussion

### 5.1 Why this case matters surgically

Our patient was a previously healthy 24-year-old woman with none of the classical predisposing factors, yet intraoperative inspection and macroscopic pathology confirmed a redundant sigmoid colon, a probable dolichosigmoid, with a long, narrow-based mesentery as the structural substrate (1, 6). The following two features make this case instructive for surgeons: the bowel was viable at both endoscopy and laparotomy, with histology showing reversible ischemic-type change rather than transmural necrosis; and definitive treatment was achieved through a planned, staged, minimally invasive pathway without a stoma. These circumstances allow the operative decisions discussed below to be examined against the patient's actual presentation.

### 5.2 Decision-making: two-step versus one-step management

In a hemodynamically stable patient without peritonitis, perforation, gangrene, or radiological ischemia, the WSES-endorsed approach is endoscopic decompression and detorsion followed by definitive sigmoid resection rather than immediate emergency surgery (1, 2). Flexible endoscopic detorsion permits direct mucosal assessment of viability, and non-operative decompression is successful in 70%–91% of patients (1). The recurrence rate after endoscopic decompression alone has been reported to be between 33% and 70% in the largest single-center series, in which elective sigmoid colectomy carried the lowest recurrence and mortality (11). Because detorsion alone is followed by recurrence in 45%–71% of patients, definitive sigmoid colectomy is recommended during the same admission once the patient is stable (1, 10). Conversely, immediate one-step surgery is indicated when there is hemodynamic instability, peritonitis, suspected or confirmed ischemia, gangrene, perforation, failed endoscopic detorsion, or non-viable bowel (1, 4, 6). Our patient fell clearly into the first group: she was stable, had no peritonitis, no CT evidence of ischemia or perforation, and showed healthy mucosa at colonoscopy, making staged management appropriate. Detorsion was achieved without complication, and a rectal decompression tube allowed safe interval planning of the definitive resection (2).

### 5.3 Hartmann's procedure versus primary colorectal anastomosis

When resection is required, the choice between a single-step resection with primary colorectal anastomosis and a Hartmann's procedure should be individualized to the patient's overall condition and intraoperative findings, particularly the presence of fecal contamination and the perfusion of the remnant colon; because the underlying pathology is benign, a full oncological resection is not required (1). A Hartmann's procedure with end colostomy is favored in an unstable or septic patient, when the bowel is gangrenous or perforated, when there is fecal contamination, or whenever an anastomosis is judged unsafe (1, 4). This is illustrated by a reported adolescent with an ischemic, 360° sigmoid volvulus after failed endoscopic decompression who underwent an open Hartmann's procedure (4). Primary colorectal anastomosis is reasonable in a stable patient with viable bowel, healthy margins, good perfusion, and a tension-free join; in large pooled data, resection with anastomosis was the most frequent procedure (96.5%) and was most often performed for uncomplicated volvulus after endoscopic detorsion (97.9%) (12). Furthermore, primary anastomosis has been used successfully, even in the emergency adolescent setting, when the bowel was viable (3, 5). Laparoscopic resection has, moreover, been associated with fewer postoperative complications than open surgery (odds ratio of 0.49) (12). In our patient, laparoscopic-assisted sigmoidectomy with an extracorporeal stapled colorectal anastomosis was appropriate because the bowel was viable, there was no contamination, the anastomosis was tension-free, and the patient was young and stable (1, 12).

### 5.4 When to create a diverting stoma

Whether to add a protective stoma depends on the assessed safety of the anastomosis. The WSES guidance frames the choice between a primary colorectal anastomosis, a defunctioned (diverted) anastomosis, or an end colostomy as an individualized decision based on the overall condition of both the patient and the colon, noting that stoma creation is not usually required in the non-emergency setting, whereas an end colostomy is most appropriate for the hemodynamically unstable or otherwise high-risk patient (1). In general colorectal practice, diversion is considered when an anastomosis is low, under tension, or poorly perfused or in the presence of contamination, gross bowel edema, unstable physiology, or significant comorbidity, none of which applied here. A diverting stoma was therefore not required because the operation was elective after decompression, the bowel was viable, and the anastomosis was tension-free and well-perfused (1).

### 5.5 Technical anastomotic considerations

The present case used an extracorporeal stapled colorectal anastomosis. Comparative, sigmoid-volvulus-specific data on stapled vs. hand-sewn and on one-layer vs. two-layer anastomosis were not found among the available references; the reviewed volvulus series and the laparoscopic meta-analysis record the procedures performed but do not compare these technical choices (1, 12). The decision is therefore generally guided by established colorectal surgical principles, namely, tissue quality, the level of the anastomosis, bowel caliber, surgeon experience, and intraoperative assessment, rather than by volvulus-specific evidence. One relevant technical caution is that a sigmoid volvulus with a markedly dilated caliber can create a size mismatch that makes a stapled end-to-end anastomosis difficult (12). We did not identify evidence in the cited references establishing the superiority of any one technique or quantifying postoperative anastomotic stricture rates after resection for sigmoid volvulus, and we therefore make no such claim.

### 5.6 Postoperative follow-up after sigmoid volvulus surgery

A fixed surveillance schedule after sigmoid volvulus surgery is not defined in the available literature, which emphasizes preventing recurrence through definitive resection rather than prescribing follow-up intervals (1, 10). In our patient, follow-up was planned for 4–6 weeks and documented at 4 weeks; a postoperative surgical clinic review at this interval is reasonable to assess wound healing, return of bowel function, abdominal symptoms, early recurrence, and any features of anastomotic complications. For the enterobiasis component, follow-up included clinical assessment, microscopic/parasitological testing, stool microscopy, and perianal adhesive-tape examination to document clearance, consistent with the cure achieved after mebendazole administration in the reported family enterobiasis (7). Repeat testing at the follow-up visit was negative. Routine colonoscopy after benign sigmoid volvulus resection is not clearly mandated solely because of the anastomosis; colonoscopy is appropriate if symptoms occur, if the colon was not adequately evaluated, or if there are alarm features such as bleeding, anemia, obstructive symptoms, weight loss, or suspicion of stricture or neoplasm. Radiological follow-up is similarly symptom-driven; for example, CT or a water-soluble contrast study should be performed if an anastomotic leak, obstruction, stricture, or recurrence is suspected. Finally, an increased malignancy risk specifically at a benign colorectal anastomosis after resection for sigmoid volvulus has not been established: no direct evidence within the cited references indicates such a risk. Cancer surveillance should therefore follow standard age- and risk-based colorectal cancer screening rather than being driven by the anastomosis itself.

### 5.7 Enterobiasis: an incidental, not causal, finding

A single adult gravid *E. vermicularis* was retrieved at colonoscopy and confirmed parasitologically by the demonstration of characteristic ova within the gravid female, supported by the family history of confirmed sibling enterobiasis (7). In adults, enterobiasis is usually asymptomatic or causes perianal symptoms and is frequently an incidental finding rather than a cause of mechanical obstruction (8); local Saudi data place it within the parasitic spectrum at low prevalence (9). There is no evidence that *E. vermicularis* causes sigmoid volvulus, and we therefore regard the infection as an incidental but parasitologically confirmed coexisting finding, valuable chiefly as an opportunity for anthelmintic treatment and household public health measures rather than as a contributory factor (7–9).

### 5.8 Strengths and limitations

The strengths of this report are the completeness of its clinical, radiological, endoscopic, intraoperative, and histopathological documentation and the demonstration of a contemporary stepwise pathway from imaging diagnosis through endoscopic decompression to elective laparoscopic resection with histologically viable margins. The follow-up period was limited to 4 weeks; therefore, late anastomotic stricture, late recurrence, and long-term bowel function could not be assessed. Longer clinical follow-up, with colonoscopy or imaging guided by symptoms or alarm features, would provide stronger postoperative outcome data. In addition, the malignancy risk at the anastomotic site after benign volvulus surgery has not been established from the available evidence, and the species was confirmed parasitologically without molecular (genotypic) testing. None of these limitations alters the central management lessons derived from this case.

## 6 Patient perspective

When invited to reflect on her illness, the patient reported that her initial symptoms seemed mild and resembled those previously experienced by her younger sister, making the sudden deterioration and need for urgent hospital assessment unexpected. She stated that explanations of the diagnosis and treatment plan helped her understand the need for endoscopic decompression followed by surgery. She was pleased with her recovery, particularly that definitive treatment was achieved without stoma formation, and reported a return to normal daily activities after discharge. She expressed appreciation for the care received and agreed that sharing her case may help raise awareness of sigmoid volvulus as a possible diagnosis in young patients with compatible symptoms. The patient and her family expressed satisfaction with the care received and consented to the publication of this report.

## 7 Conclusion

Sigmoid volvulus should be considered in the differential diagnosis of acute large-bowel obstruction in young adults, even in the absence of classical risk factors. Contrast-enhanced CT demonstrating the whirl sign and an abrupt point of collapse remains the diagnostic linchpin. In hemodynamically stable patients without features of ischemia, perforation, or peritonitis, a two-step pathway of endoscopic detorsion followed by elective laparoscopic sigmoidectomy during the same admission is a safe and effective strategy that minimizes stoma utilization, reduces recurrence, and preserves bowel function. The intraoperative-equivalent identification of a parasitologically confirmed *E. vermicularis* adult in this case represents an incidental coexisting infection rather than a causative or contributory factor in the volvulus; nonetheless, the finding justified anthelmintic therapy and household-level public health advice and reinforces the value of thorough endoscopic inspection during decompression.

## Data Availability

The original contributions presented in the study are included in the article/Supplementary Material, further inquiries can be directed to the corresponding author.
