# Supplementary figures and images for: Sigmoid volvulus and incidental enterobiasis in a young adult: a case report
Source: Front Surg. 2026 Jul 6;13:1883256. doi: 10.3389/fsurg.2026.1883256 (PMC13382634; doi:10.3389/fsurg.2026.1883256)

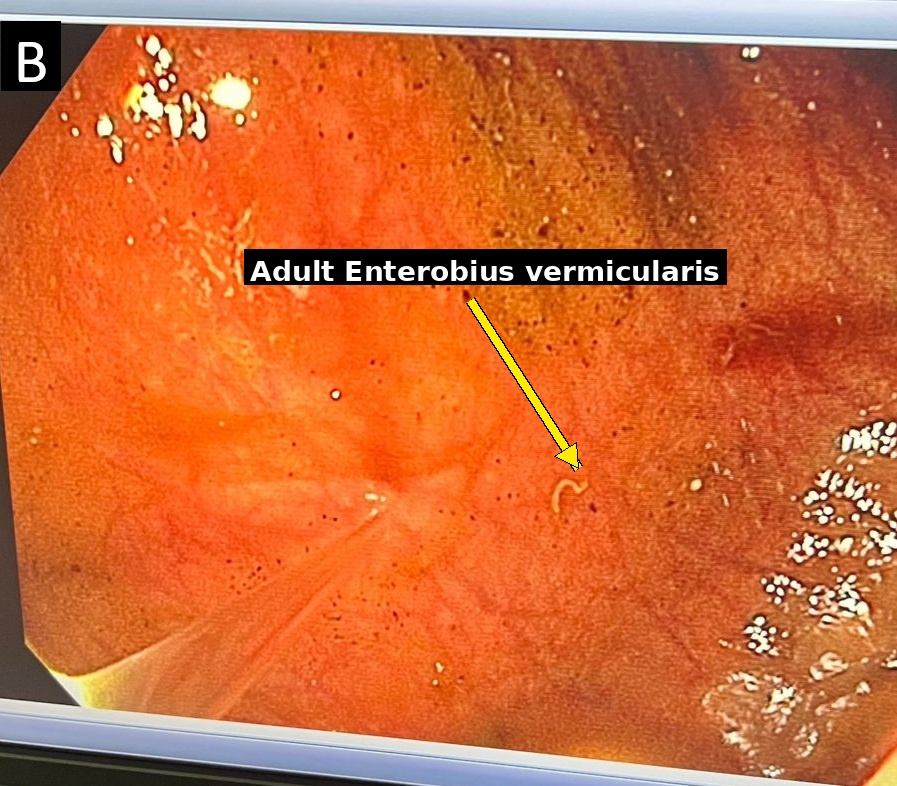

Supplement: Supplementary file 1 [file Image1.jpeg]

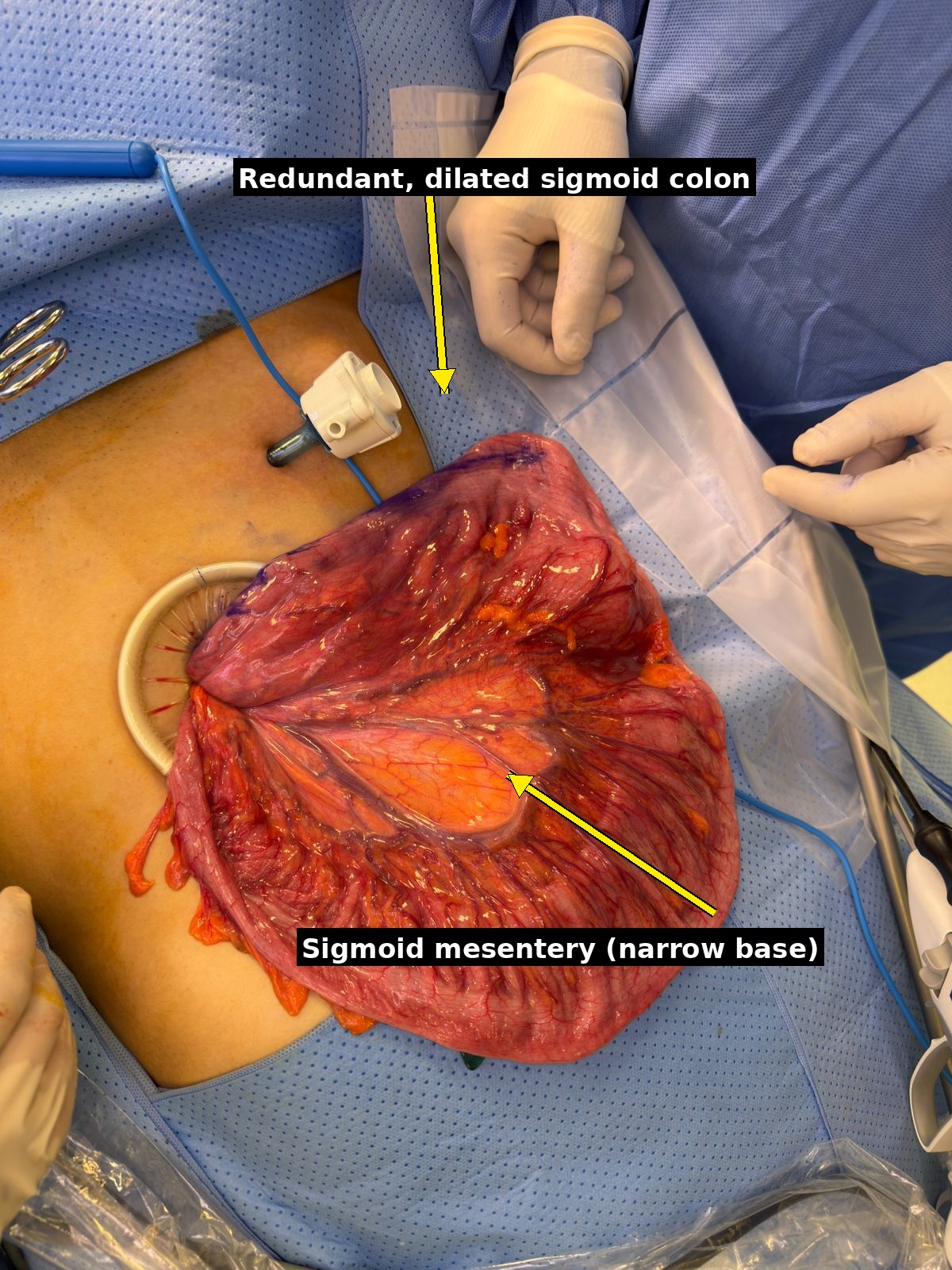

Supplement: Supplementary file 2 [file Image2.jpeg]
